# Changes in phospholipid metabolism in exosomes of hormone-sensitive and hormone-resistant prostate cancer cells

**DOI:** 10.7150/jca.48906

**Published:** 2021-03-15

**Authors:** Xianlin Yi, You Li, XiaoGang Hu, FuBing Wang, Tiangang Liu

**Affiliations:** 1 Department of Urology, The Affiliated Cancer Hospital of Guangxi Medical University & Guangxi Cancer Research Institute, Nanning 530021,China.; 2 Key Laboratory of Combinatorial Biosynthesis and Drug Discovery, Ministry of Education and School of Pharmaceutical Sciences, Wuhan University, Wuhan 430071, PR China.; 3 Life science institute of East China Normal University, Shanghai 200241, P.R. China.; 4 Department of Laboratory Medicine, Zhongnan Hospital of Wuhan University, Wuhan 430071, P.R. China.; 5 Wuhan infectious diseases and cancer research center, Chinese Academy of Medical Sciences, Wuhan 430071, P.R. China.; 6 Hubei Engineering Laboratory for Synthetic Microbiology, Wuhan Institute of Biotechnology, Wuhan 430075, PR China.

**Keywords:** lipidomics, exosome, prostate cancer

## Abstract

**Background:** To explore the changes in lipids in exosomes of hormone-sensitive and hormone-resistant prostate cancer cells and develop an inexpensive and rapid technique for screening lipid-based biomarkers of prostate cancer.

**Methods:** Exosomes were extracted from LnCap, PC_3_ and DU-145 cells, and their lipid composition was analyzed quantitatively using high-throughput mass spectrometry. Exosomes released by LnCap prostate cancer cells were also purified using a modified procedure based on polyethylene glycol (PEG) precipitation.

**Results:** Exosomes extracted from LnCap cells contained higher proportions of phosphatidyl choline, phosphatidyl ethanolamine and phosphatidyl inositol lipids than whole LnCap cells. Lysophosphatidylcholine, a harmful intermediate product of phosphatidylcholine metabolism *in vivo*, was not found in LnCap cells but in exosomes. Phospholipids were different in exosomes from LnCap, PC3 and DU-145 prostate cancer cells. The main lipid pathways involved, i.e., glycerophospholipid metabolism, autophagy, and ferroptosis pathways, were also different in these cells. Exosomes isolated by this modified PEG precipitation technique were similar in purity to those obtained using a commercial kit.

**Conclusions:** This study demonstrates that phosphatidylcholine and its harmful product lysophosphatidylcholine may play important roles in hormone-sensitive prostate cancer. Phospholipid exosome metabolism was changed in hormone-sensitive and hormone-resistant prostate cancer cells. The LPC, lipid pathway of autophagy and ferroptosis may act as therapeutic targets. The possibility of purifying prostate cancer cell exosomes using modified PEG precipitation is suitable for cancer screening.

## Introduction

Prostate cancer (PCa) is the most common solid malignant disease worldwide 1, and in advanced patients, the first line of treatment is androgen deprivation therapy (ADT). However, ADT can cause metabolic syndrome and lipid-specific changes, such as high total cholesterol and triglycerides 2. Epidemiological studies have indicated that a Western diet can cause the progression of PCa to a lethal disease 3. Recent studies have suggested that lipids can exacerbate fatal prostate cancer in mouse models 4, and analyses of lipids in blood and urinary exosomes have identified lipid species differing significantly between healthy controls and prostate cancer patients 5-7.

Exosomes are lipid bilayer-enclosed nanoparticles that are nearly as large as viruses and serve as “garbage bags” for debris and waste released from cells. These particles have become quite attractive as a potential source of cancer biomarkers since they often contain tumor-specific molecules 1, 7. The membranes of exosomes contain a variety of lipids, although relatively little is known about their composition compared with their cell proteomics and transcriptomics 8.

Studying exosomes released by prostate cancer cells may provide valuable biomarkers for diagnosing this disease and designing effective therapies 9. Prostate cancer, the most common malignancy in men in the US, is diagnosed most accurately by invasive biopsy, which can nevertheless underestimate the pathological grade or completely miss the tumor tissue because of the heterogeneous, multifocal nature of the disease 10. Less invasive diagnostic testing based on elevated levels of prostate-specific antigen (PSA) can identify up to 60% of cases of early prostate cancer, although the test gives false negative results in approximately 30% of patients because of its relatively low specificity 11, 12. Identifying more reliable biomarkers may help clinicians distinguish benign prostate disease from prostate cancer, reduce unnecessary prostate biopsies, and design more effective treatments 13. Biomarkers in exosomes in the blood or urine could be monitored noninvasively in a screening suite, thereby reducing costs and risks associated with biopsy 14.

Lipid metabolism supports PCa cell growth and ADT resistance. However, whether this change occurs before or after androgen receptor reprogramming is still unknown 2. Research into lipid-based biomarkers remains in the early stages because the characteristics and mechanisms of action of lipids in prostate cancer remain obscure. Therefore, in the present study, we applied mass spectrometry to comprehensively examine potential lipid biomarkers of three prostate cancer cell lines with different degrees of malignancy.

As part of this work, we sought to devise an efficient and relatively straightforward exosome purification protocol because existing methods rely on ultracentrifugation, other size-based fractionation methods, microfluidics or immunoaffinity-based capture, which generally involve costly equipment or reagents or lengthy durations 1, 15. Therefore, we developed an inexpensive procedure for extracting exosomes using modified polyethylene glycol (PEG) precipitation. We validated the effectiveness of our protocol by comparing it with two commercially available exosome preparation kits.

## Methods

### Cell culture

The prostate cancer cell lines LnCap, PC3 and DU145 were purchased from the Wuhan University Strain Preservation Center (Wuhan, China) and cultured in Ham's F12 medium or Dulbecco's modified Eagle's medium (Corning, US). These cells were supplemented with exosome-free serum Ham's F12 medium or Dulbecco's modified Eagle's medium supplemented with exosome-free serum (Corning, US), 2 ng/mL basic fibroblast growth factor (Corning, US), 2 mM glutamine (Yongjin Biotech, Guangzhou, China), and 10 units/ml penicillin/streptomycin. Cultures were maintained at 37 °C in an atmosphere containing 5% CO_2_.

### Exosome extraction using PEG precipitation

PEG solution was prepared by mixing 16 g PEG (Wuhan Chemical, Wuhan, China) with 5.844 g NaCl in a final volume of 100 ml water. PEG solution (1 ml) was mixed with cell cultures and centrifuged at 500 *g* and then at 2000 *g* to remove debris when needed.

The prostate cancer cells were resuspended, and 1 volume of PEG solution was added. The mixture was incubated overnight at 4 °C and centrifuged at 16000 *g* for 1 h. The pellet was suspended in phosphate-buffered saline (PBS), and the cells were pelleted again for 1 h and finally resuspended in fresh PBS.

### Exosome extraction using the Total Exosome Isolation Reagent Kit

Prostate cancer cells were centrifuged at 2000 *g* for 30 min, suspended in 1 mL medium, and supplemented with 0.5 mL of reagent from the Total Exosome Isolation Reagent Kit® (Thermo Fisher Scientific, US). Samples were centrifuged at 10,000 *g* for 1 h at 2-4 °C, and the pelleted exosomes were suspended in 0.1 ml PBS. The resuspended exosomes were stored for up to 1 week at 2-8 °C or for longer periods at -20 °C or colder.

### Exosome extraction using the exoEasy Maxi Kit

Cells were centrifuged at 16,000 *g* for 10 min. The exosomes were isolated using the exoEasy Maxi Kit (QIAGEN®, Germany). The suspension was mixed with XBP buffers and transferred to an affinity spin column, which was centrifuged at 600 *g* for 50 s. XWP buffer (3.5 ml) was added, and the column was centrifuged for 5 min at 5000 *g.* The eluate (700 μl) was added to a QIAzol membrane and centrifuged at 5000 *g* for 5 min.

### Protein assay of exosomes

The total protein concentration of exosomes purified by the three methods was determined using the Pierce® BCA protein assay kit (Thermo Fisher Scientific).

### Transmission electron microscopy of exosomes

Exosomes prepared using the three methods (10 μl) were transferred to copper grids, fixed with 4% glutaraldehyde (15 μl, Wuhan Chemical) for 5 min, and negatively stained using sodium phosphotungstate solution. Then, stained grids were examined using a 200-kV Tecnai G2 transmission electron microscope.

### Western blotting of exosome proteins

Exosomes were denatured by boiling in cell buffer and fractionated by SDS-PAGE (Gibco). Proteins were transferred to membranes, which were incubated with rabbit monoclonal antibodies (CST, US) against either androgen receptor or PSA overnight at 4 °C. Then, membranes were incubated with labeled goat anti-IgG antibody (CST) at room temperature for 2 h.

### Light scattering of exosomes

Exosomes isolated by the three methods were analyzed in a dynamic light scatterer (ALV-CGS-3, ALV-Laser Vertriebsgesellschaft m-b. H, Germany).

### Lipid extraction from cells

LnCap prostate cancer cells were harvested and suspended, and then 5 volumes of quenching buffer (quenching solution: 60% (v/v) analytical grade methanol, 0.85% (w/v) ammonium bicarbonate (pH 7.4) were added to a 50-mL conical tube that had been precooled to -4 °C. The mixture was centrifuged at 1000 *g* for 1 min, and the pellet was resuspended in 200 μl methanol precooled to -80 °C. The resuspension was stored at -80 °C until analysis.

### Lipid extraction from exosomes

Exosomes (0.5 ml) were added to chromatography-grade methanol and vortexed for 5 min. Then, dichloromethane was added, the mixture was vortexed for 5 min, ddH_2_O was added, and the mixture was vortexed for 30 s. The mixture was centrifuged at 2000 rpm at room temperature for 1 min, and the lower organic layer was vacuum-dried at room temperature for subsequent analysis.

### Mass spectrometry and bioinformatics analysis of lipids

Lipid extracts were fractionated on a Shimadzu ODS-3 C18 column at 40 °C and subjected to triple quadrupole mass spectrometry (QTRAP, AB SCIEX). Mass spectra were analyzed using LipidView software (AB SCIEX, US). Prior to the experimental runs, the spectrometry conditions were optimized based on the peak areas of a phosphatidylcholine standard. The fragmentation voltage was optimized by testing in the range 60-240 V in 20-V intervals, and the collision energy was set so that the strongest daughter ion response was 5-40 eV in second-order mass spectra. The lipid pathways were analyzed using a Lipid Pathway Enrichment Analysis (LIPEA) (https://lipea.biotec.tu-dresden.de/analyze), Biotechnology Center (BIOTEC). Technische Universität Dresden. Tatzberg 47/49. 01307, Dresden. Germany.

## Results

### Extraction of lipids from isolated exosomes and whole LnCap cells

**Figure 1:**
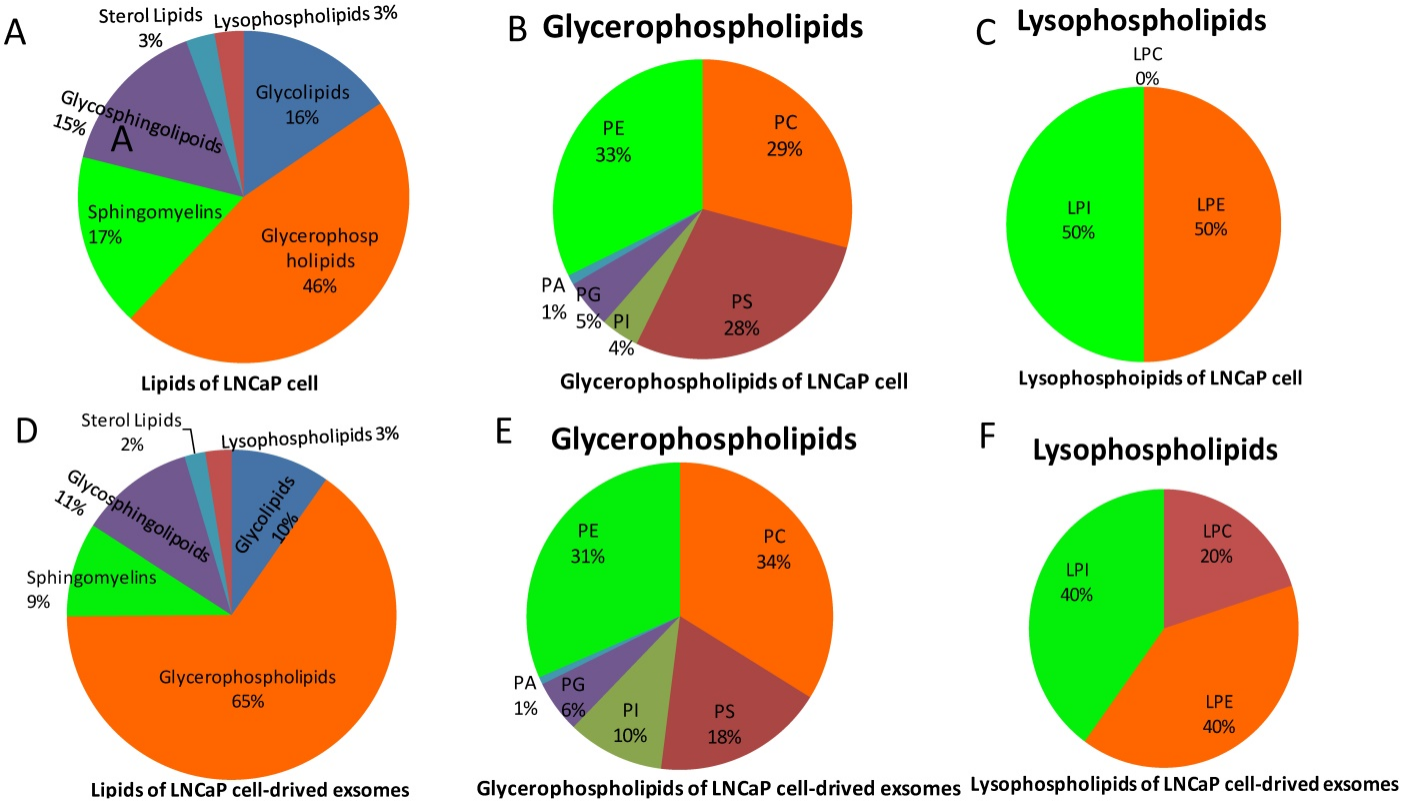
Lipid classes of LnCap cells and exosomes in this study. **A,** Lipid species of LnCap cells in this study. **B,** Composition of glycerophospholipids in LnCap cells. **C,** Composition of lysophospholipids in LnCap cells. **D,** Lipid species of LnCap cell-derived exosomes. **E,** Composition of glycerophospholipids in LnCap cell-derived exosomes. **F,** Composition of lysophospholipids in LnCap cell-derived exosomes.

Extraction of lipids from isolated exosomes as well as whole LnCap prostate cancer cells (Figure 1, supplement Figure 1) and then analysis by high-resolution mass spectrometry revealed few differences in the constituents of sphingomyelin or glycoside ceramide. In contrast, the constituents of phosphatidylcholine varied substantially between exosomes and cells (Figure 1B, E). More interestingly, lysophosphatidylcholines (LPCs), which are harmful intermediate products of phosphatidylcholine metabolism *in vivo*, were not found in LnCap cells but in exosomes (Figure 1C, F). We also noticed that there were no great differences between the species of glycosphingolipoid as a whole and among its components (CER, HEXCER, LACCER) in LnCap cells and exosomes. Furthermore, our study also shows that LPCs were not found in exosomes extracted from PC3 and DU-145 cells, which represents hormone-resistant PCa (Figure S2). The detected intensities were defined by ion mass/charge (m/z) (Figure S1).

### Lipid Pathway Enrichment Analysis of exosomes of LnCap, PC3, and DU-145 cells

Lipids extracted from isolated exosomes of LnCap, PC3 and DU-145 prostate cancer cells were analyzed, and the results showed that phospholipids were also different in the three prostate cancer cell lines that represent different malignancies. Glycerophospholipids were the most abundant lipids in LnCap cell-derived exosomes, while sphingolipids were the most abundant lipids in PC3 and DU-145 cell-derived exosomes. Glycerophospholipids and lysophospholipids were also different between the lipids of LnCap, PC3 and DU-145 cell-derived exosomes (Figure S2).

**Figure 2:**
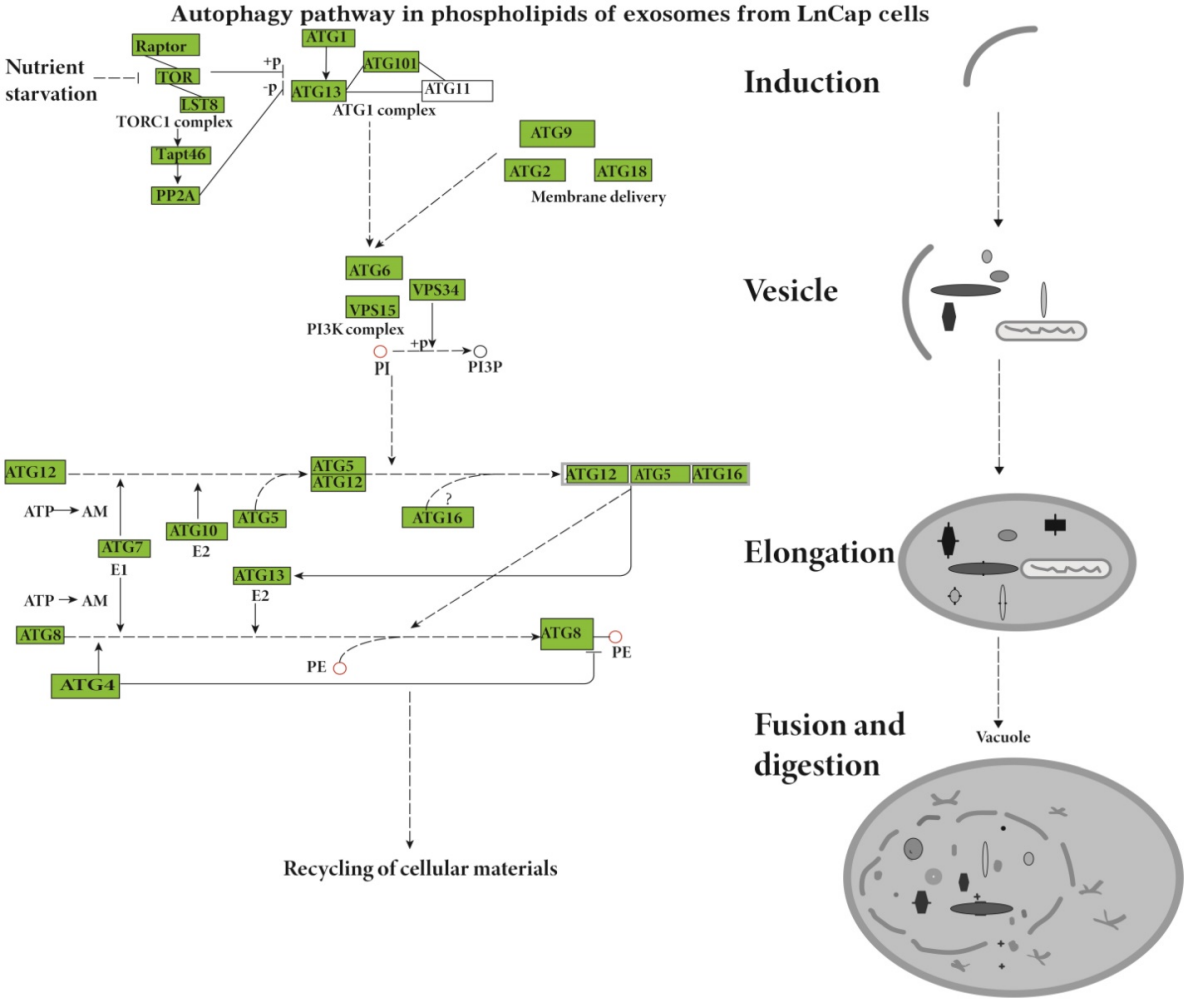
Involvement of the autophagy pathway in the phospholipids of exosomes from LnCap cells (Lipid Pathway Enrichment Analysis, LIPEA). Copyright from the Lipid Pathway Enrichment Analysis (LIPEA). Created by Biomedical Cybernetics Group, https://lipea.biotec.tu-dresden.de/analyze.

**Figure 3:**
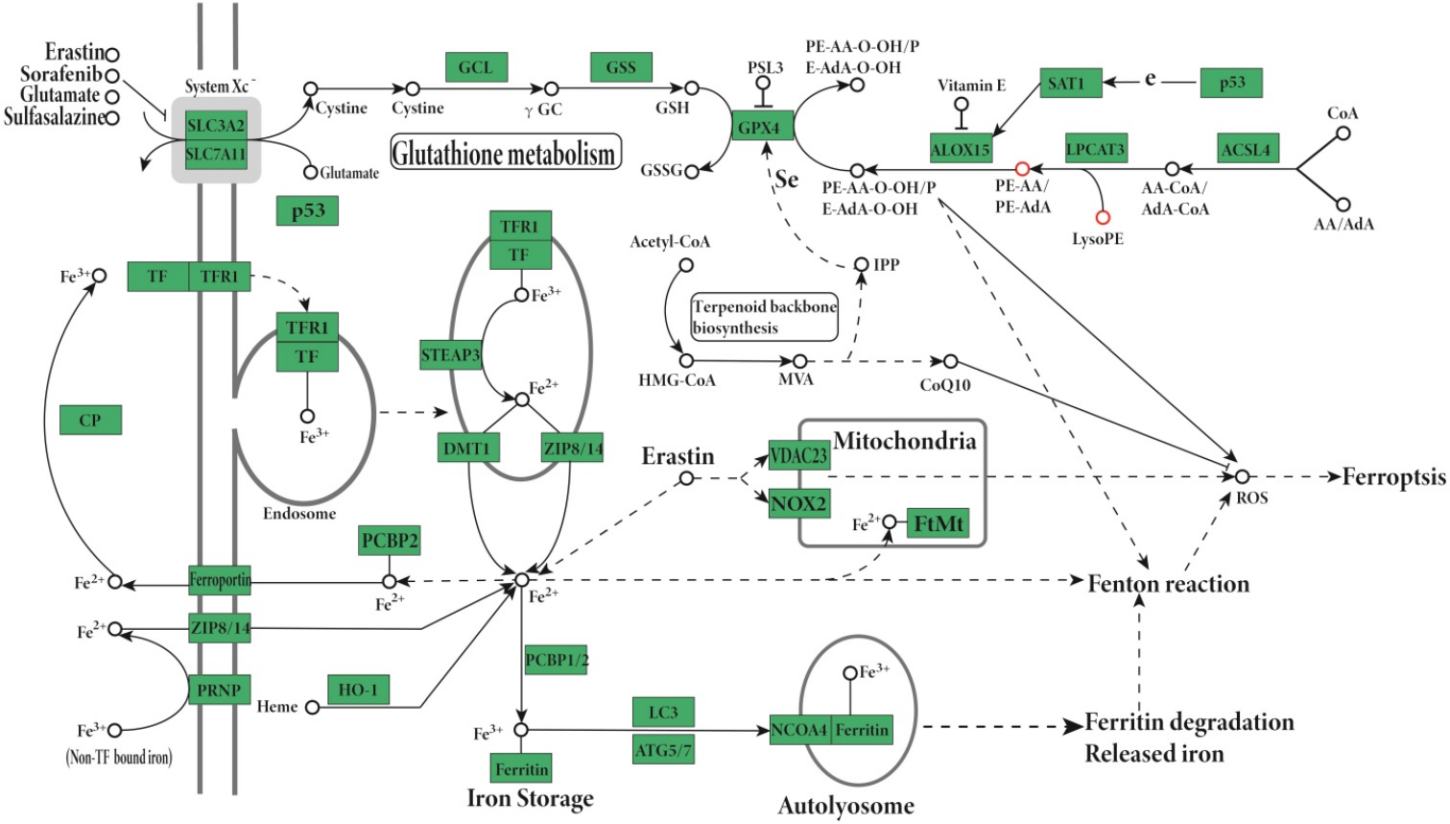
Lipid Pathway Enrichment Analysis (LIPEA) has shown that the autophagy pathway is involved in the phosphorylation of exosomes from PC3 and DU-145 cells. Copyright from Lipid Pathway Enrichment Analysis (LIPEA).

The Lipid Pathway Enrichment Analysis (LIPEA) showed that glycerophospholipid metabolism (Supplement Figure 3), autophagy (Figure 2), and ferroptosis (Figure 3) pathways were different in these cells (Table 1). All of the figures in our manuscript were reedited based on the database diagram from the Lipid Pathway Enrichment Analysis (LIPEA). The website, which was created by Aldo Acevedo et al., is at https://lipea.biotec.tu-dresden.de.

### Extraction of exosomes using modified PEG precipitation and commercial kits

**Figure 4:**
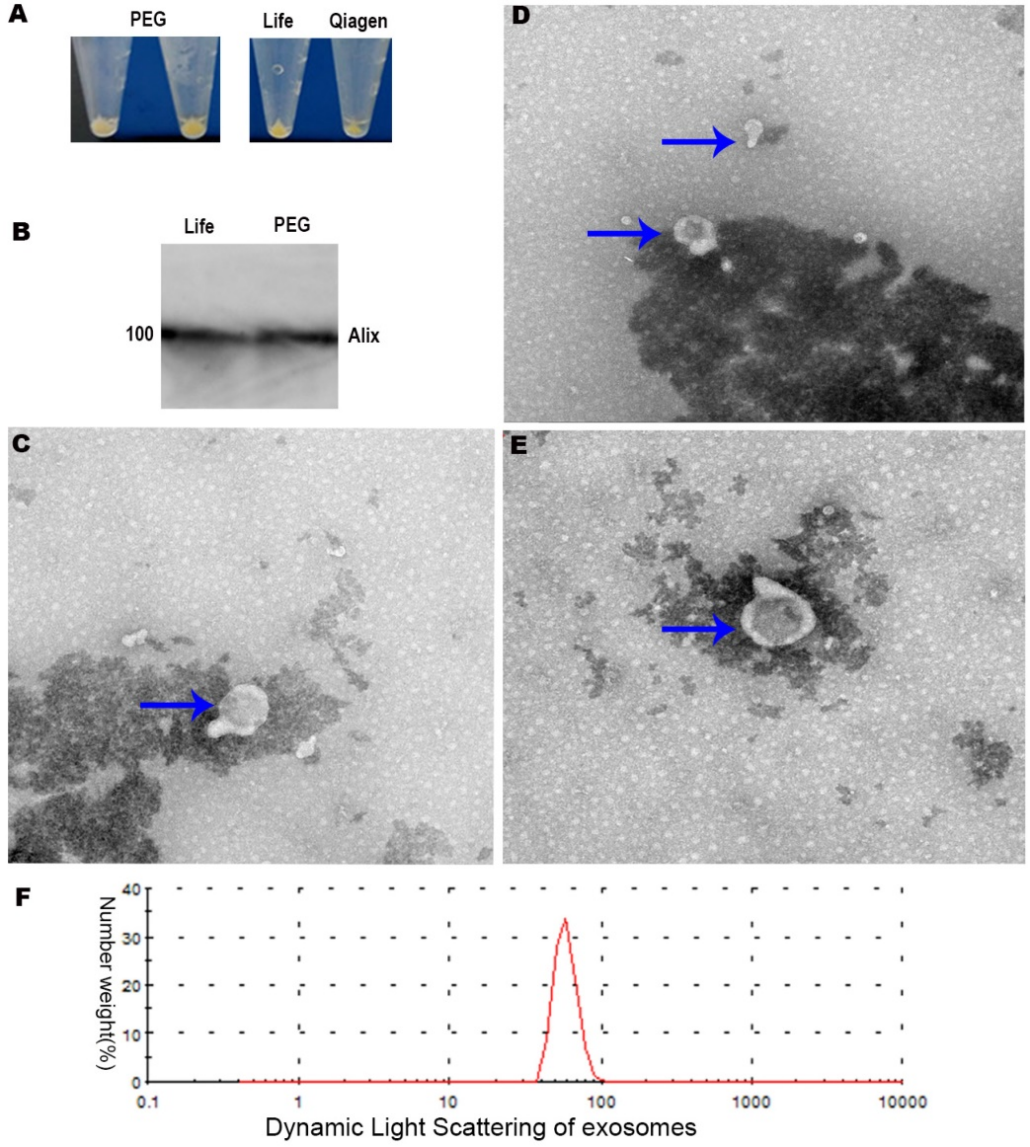
** A,** Exosomes extracted by three methods. **B,** Western blot results of exosomes. **C,** LIFE Kit® method's result of electron microscope (200 KV, X19000, 200 nm). **D,** QIAGEN Kit® method's result of electron microscopy. **E,** PEG precipitation method's result of electron microscopy. **F,** Dynamic light scattering results of exosomes extracted by Life's Kit®.

We purified exosomes using two commercial kits as well as our modified procedure based on PEG precipitation (Figure 4A). Transmission electron microscopy showed that exosomes prepared by commercial approaches were membrane-enclosed and semispherical with a saucer shape and a concave side (Figure 4C-E). Exosomes prepared using our PEG method were similar in morphology and homogeneity (Figure 4A-B). The vast majority of exosome diameters ranged between 30 and 120 nm (Figure 4E). In terms of the background of transmission electron microscopy, the background and morphology of exosomes of the modified PEG group were similar to those of the two commercial kits.

The identification and purity of the exosome preparations were confirmed by Alix, the molecular markers of the exosomes related to multivesicular body synthesis (Western blotting, Figure 4B). The desired protein patterns were present in exosomes isolated using the LIFE kit® and QIAGEN Kit® as well as our PEG method (Figure 4B). The electron microscopy and Western blotting results were similar for exosomes purified using the Total Exosome Isolation kit or our PEG method (Figure B-E). Dynamic light scattering revealed that exosomes extracted by the LIFE Kit® were consistent with the literature 16, as shown in Figure 4F. Subsequently, the isolated exosomes extracted by the LIFE Kit® were used for the lipid analysis, and the Lipid Pathway Enrichment Analysis showed that there were differences among isolated exosomes of LnCap cells and PC3, DU-145 and LnCap prostate cancer cells 17.

## Discussion

Although most cancers employ glycolysis as the main source of energy, PCa prefers to employ lipid metabolism instead 2. A prospective multicenter study proved that the metabolite profile was related to the risk of more aggressive PCa. In this study, the lipids of exosomes extracted from three different PCa cells were investigated. The phospholipids of these PCa cells are different, and their lipid pathways are also different.

We detected a greater diversity of phosphatidylcholine, phosphatidylethanolamine and phosphatidylinositol between exosomes derived from LnCap prostate cancer cells and intact LnCap cells, which is consistent with a previous study in which the phosphatidylcholine and phosphatidyl ethanolamine composition of exosomes from urine differed substantially between prostate cancer patients and healthy controls 3, 18. Several studies have confirmed that phosphatidylcholines are associated with more aggressive risk and more advanced PCa 19. Moreover, recent evidence indicates that phosphatidylcholine was increased in androgen-responsive LNCaP and 22RV1 cells. In contrast, phosphatidylcholine is significantly reduced in hormone-nonsensitive DU-145 cells and nonmalignant PNT1a cells 20. These findings may reflect the apparent association between phosphatidylcholine metabolism and prostate cancer tumorigenesis and development 21. In addition, serum levels of phosphatidylinositol are strongly associated with prostate cancer aggressiveness 22.

The study results indicate that the lipid pathways of phospholipids are different in hormone-sensitive and hormone-nonsensitive PCa cells. The major lipid pathways involved in phospholipids of exosomes of prostate cancer cells are glycerophospholipid metabolism (Figure S3-S4), autophagy (Figure 2), and ferroptosis (Figure 3) pathways (Table 1). More phospholipids were involved in the autophagy pathway in hormone-sensitive LnCap cells than in hormone-nonsensitive PC3 and DU-145 cells, and the latter was involved more in ferroptosis. Autophagy not only regulates lipid metabolism but also suppresses tumorigenesis and metastasis 23. Failed autophagy is necessary for the initiation of cancer. Studies have suggested that autophagy may play a dual role according to the stage of prostate cancer 24-26. In the early stages (hormone sensitivity), the induction of autophagy may increase cell death 27. However, the late stage of prostate cancer (hormone-nonsensitive) may exploit autophagy to protect cancer cells, reduce the damage of nutrient stress or chemotherapy, and meet the continuous consumption of tumor survival and rapid proliferation 24-26.

Ferroptosis, an iron-dependent form of nonapoptotic cell death, is occasionally induced by lipid peroxidation 28. Prostate cancer mainly relies on fatty acid β-oxidation to obtain energy 29, whereas ferroptosis can catalyze the high expression of unsaturated fatty acids, which leads to extensive lipid peroxidation and cell death. A recent study showed that LNCaP cells were highly iron-sensitive while DU-145 and PC 3 cells were poorly iron-sensitive. The toxicity of iron mainly drives lipids to promote ferroptosis 30.

Lysophosphatidylcholines (LPCs) are correlated with inflammation 31, oxidative stress, insulin resistance, apoptosis 32, lipid remodeling and signaling lipid generation 33. LPCs are biomarker of some tumours, including prostate cancer 14. Multiple LPC species are decreased in renal cell carcinoma and significantly associated with lung cancer 18.

More importantly, LPCs are associated with the recurrence and progression of prostate cancer. LPCs of prostate tissue are independent predictor of biochemical recurrence in patients underwent radical prostatectomy 34. LPCs are different in prostate cancer compared with benign prostate epithelium 34.

However, LPCs, which are harmful intermediate products of phosphatidylcholine metabolism *in vivo*, were not found in LnCap cells but in exosomes (Figure 1).

Higher abundances of preoperative lysophosphatidylcholines in blood indicate recurrence in patients with radical prostatectomy 35. However, studies have shown that higher levels of plasma lysophosphatidylcholines are related to lower risks of prostate cancer 21. Recently, a prospective study of the European Prospective Investigation into Cancer and Nutrition (EPIC) confirmed that higher concentrations of blood phosphatidylcholines before diagnosis were associated with aggressive PCa risk. Meanwhile, higher blood lysophosphatidylcholines indicate a lower risk of advanced stage prostate cancer at diagnosis and a better prognosis 19.

These inconsistent results are associated with the type of specimen used, the degree of malignancy and the stage of cancer; moreover, they suggest that phospholipids such as LPC/LPI of exosomes may act as a specific target for PCa treatment. LPC and lysophosphatidylinositol (LPI) promote the migration of prostate cancer cells 36.

The other reason for these inconsistent is the class of LPC. In the current study, the main LPC is 20:1 LPC, which only showed in the exosomes derived from LnCap cells (Figure 1, Supplement Figure 2). The 20:1 LPC of exosomes may plays a role in the inhibition of prostate cancer cell metastasis, while 17:0 LPC, 20:3 LPC and 20:4 LPC are positively associated with risk of aggressive prostate cancer in plasma 37, In another study, the higher plasma levels 18:0 LPC is related to lower risks of prostate cancer 38. The expression of 16:0 LPC has been reported to be lower in PCa than in benign prostate tissue 34. Deceased expression of 16:0 LPC in PCa tissue can independently predict biochemical recurrence after radical prostatectomy 34.

The level of LPCs are correlated with the risk of prostate in human plasma, and the phosphatidylcholine metabolism may drive tumorigenesis 38, LPC can induce biomarker production of cellular senescence, and induce DNA injury and cell canceration in cholangiocytes 39. A clinical trial of prostate cancer was conducted by Küllenberg de Gaudry et al. 40, who found that the plasma concentration of LPCs only increases significantly in patients without prostate tumors after high marine phospholipid intake. The lack of increased LPCs levels after marine phospholipid supplementation in patients with actively metastatic prostate cancer suggests that tumor cells have a higher demand for LPC 40.

Supplement of LPC can increase membrane rigidity and reduced the metastatic potential in animals 41. No evidence that LPC itself can stimulate migration, but it can undergo conversion into Lysophosphatidic acid (LPA) to promote cell invasion 42. It's been proven that LPC inhibits cancer cell invasion involves the inhibitions of LPC conversion into LPA by autotaxin via LPA1/3 receptors 42.

LPC can hydrolyze to lysophosphatidic acid (LPA), which represses autophagy in prostate cancer cells 43. The addition of LPA to serum-starved cells dramatically increased phospho-uncoordinated-51-like kinase 1 in Du-145 and PC3 cells but not in LNCaP cells 43. The prostate cancer cells that are distressed and undergoing nutrient deprivation, LPA may act as a critical molecule that protects prostate cancer cells from autophagic cell death.

The LPCAT family plays a dominant role in the reacylation of lysophospholipids 28. Overexpression of lysophosphatidylcholine acyltransferase (LPCAT) 1 promotes the initiation and progression of renal cell carcinoma, which may occur through the conversion of LPC to PC.LPCAT 1, the catalytic enzyme of phosphatidylcholine, is also an independent predictor of a high risk for biochemical recurrence of prostate cancer 44. In addition, LPCAT2 is expressed in aggressive prostate cancer 28.

The involved lipid pathways are changed in hormone-sensitive and hormone-resistant prostate cancer cells (Table 1, Figure 2-3, Figure S3-S4, Lipid Pathway Enrichment Analysis (LIPEA)). The LPCs and lipid metabolism may act as tumor-specific targets 14.

Our results are consistent with previous work in which lipids in exosomes derived from PC3 cells were found to be higher in abundance than those in whole PC3 cells 22, 45. Lipids are attractive as potential biomarkers because they can be classified according to the composition, saturation degree, and length of the fatty acid chains. These differences could help distinguish the characteristics of metabolism 46 between normal and cancerous cells 47.

Exosomes offer great advantages as cancer monitors. However, studies on exosomes from hormone-sensitive LnCap cells are currently lacking, and these cells are characterized by lymph node metastasis. In the hormone-sensitive period, castration was more effective and persistent and the patients lived longer than in CRPC. However, many patients have an unpredictable short hormone-sensitive period; therefore, advanced screening must be performed to benefit those patients' overall survival 1. Exosome-derived lipids may help to distinguish these patients and are not as sensitive. Current studies mainly focus on castration-resistant PC3 cells, which are characterized by bone metastasis 18 and represent aggressive prostatic adenocarcinoma 48.

As the main techniques for the isolation of exosomes, ultracentrifugation-based techniques and immunoaffinity capture-based techniques are associated with high equipment or reagent costs 1. Size-based techniques require dedicated equipment and moderate equipment costs. Microfluidics-based techniques lack standardized and large-scale tests on clinical samples and lack method validation 15.

In the present study, we developed a simple, inexpensive and fast method for isolating exosomes from two prostate cancer cell lines based on modified PEG precipitation. The resulting exosomes are similar to those obtained using two commercial kits.

Although PEG precipitation is not the most accurate method for purifying exosomes, the derived exosomes can retain their physiological activity 49. Our modified PEG precipitation procedure provides a rapid and inexpensive method of isolating exosomes from cancer cells via a screening suite, and it can rival commercial kits and bypass lengthy ultracentrifugation (Figure 4A-E). Normally, low-speed centrifugation is used in traditional PEG precipitation 50. In the work by Anna-Kristin Ludwig et al., HEK293T cells were centrifuged at 110,000 × g for 2 h using modified PEG precipitation methods 50; however, their technique may require ultracentrifugation.

In our study, the cell cultures were centrifuged at high speed combined with low-speed centrifugation at 500 g, 2000 g and 16000 g. A speed of 16000 g is near the maximum speed of ordinary centrifuges in many laboratories. Therefore, a special supercentrifuge may not be needed in clinical screening.

For HEK293T cells, the concentration of the highest particle yield was 10-12% for PEG 6000 and 8-10% for PEG 8000 50. For tissues, the concentration of PEG for extracting synovial tissue-derived exosomes was 8% 51.

PEG might disturb downstream applications in subsequent studies. To keep the density as low as possible, approximately 8% polyethylene glycol (PEG) was used to extract exosomes from prostate cancer cells in our study.

The next step would be to confirm that our method works for isolating exosomes from serum, urine and other secretions 52. Our modified PEG method may need further refinement, such as through incorporation of an ultracentrifugation step, since transmission electron microscopy revealed that a small proportion of our exosomes had diameters >200 nm.

A disadvantage of the current study is that liposome analysis is not a quantitative analysis and only includes cell lines but not normal prostate cell lines. Moreover, only one molecular marker (Alix) was tested in the protein content-based exosome characterization. Although the morphological characteristics of prostate cell-derived exosomes were tested by transmission electron microscopy, they were not sufficient.

The PEG method was not used to analyze the lipid composition, which was also a disadvantage. For more sophisticated and demanding liposome experiments, commercial kits may have higher uniformity. Furthermore, our PEG method has been indirectly confirmed by an applying patent (http://www.xjishu.com/zhuanli/27/202010064906_3.html).

## Conclusions

This study describes the first procedure for extracting exosomes from LnCap cancer cells using modified PEG precipitation, and the results were validated against exosomes prepared using commercial kits. The results of our novel procedure provide detailed insights into the lipidome of exosomes derived from LnCap prostate cancer cells. Phosphatidylcholine is increased in exosomes, and lysophosphatidylcholines (LPCs), which are harmful intermediate products of phosphatidylcholine metabolism *in vivo*, were not found in LnCap, PC3 and DU-145 cells but in exosomes extracted from LnCap cells. The lipid pathways of phospholipids were different in hormone-sensitive and hormone-nonsensitive PCa according to our current results. The lipid pathway of autophagy and ferroptosis may act as therapeutic targets.

## Supplementary Material

Supplementary figures and tables.

## Figures and Tables

**Table 1:** Major lipid pathways involved in phospholipids of exosomes of prostate cancer cells

LnCap			PC3			DU-145		
Pathway name	lipids	P	Pathway name	lipids	P	Pathway name	lipids	P
Glycerophospholipid metabolism	5	0.003	Glycerophospholipid metabolism	12	0.000	Glycerophospholipid metabolism	10	0.000
Sphingolipid metabolism	4	0.008	Ferroptosis	3	0.003	Ferroptosis	3	0.002
Glycosylphosphatidylinositol (GPI)-anchor biosynthesis	2	0.005	Phospholipase D signaling pathway	2	0.014	Choline metabolism in cancer	2	0.006
Steroid biosynthesis	2	0.516	Glycerolipid metabolism	2	0.063	Phospholipase D signaling pathway	2	0.012
Autophagy - animal	2	0.009	Fat digestion and absorption	2	0.019	Retrograde endocannabinoid signaling	2	0.016
Sphingolipid signaling pathway	2	0.049	Retrograde endocannabinoid signaling	2	0.019	Fat digestion and absorption	2	0.016
Autophagy - other	2	0.005	Choline metabolism in cancer	2	0.007	Glycerolipid metabolism	2	0.055
Retrograde endocannabinoid signaling	2	0.039	Pathways in cancer	2	0.063	Pathways in cancer	2	0.055
Ovarian steroidogenesis	2	0.166	Pathogenic *Escherichia coli* infection	1	0.028	Pathogenic *Escherichia coli* infection	1	0.026
Ferroptosis	2	0.071	Systemic lupus erythematosus	1	0.028	Systemic lupus erythematosus	1	0.026

Data from Lipid Pathway Enrichment Analysis (LIPEA). *Lipids: Converted lipids (number).

## Abbreviations

CHOL: cholesterol
CE: Cholesterol Ester
DAG: glyceride diester
PC: phosphatidylcholine
PA: phosphatidic acid
PE: phosphatidylethanolamine
PG: phosphatidylglycerol
PI: phosphatidylinositol
PS: phosphatidylserine
SM: sphingomyelin
LPC: lysophosphatidylcholine
LPE: lysophosphatidylethanolamine
LPI: lysophosphatidylinositol
PG: phosphatidylglycerol
