# Supplementary material for: Changes in phospholipid metabolism in exosomes of hormone-sensitive and hormone-resistant prostate cancer cells
Source: J Cancer. 2021 Mar 15;12(10):2893–902. doi: 10.7150/jca.48906 (PMC8040901; doi:10.7150/jca.48906)
Supplement: Supplementary file 1 — Supplementary figures and tables. [file jcav12p2893s1.pdf]

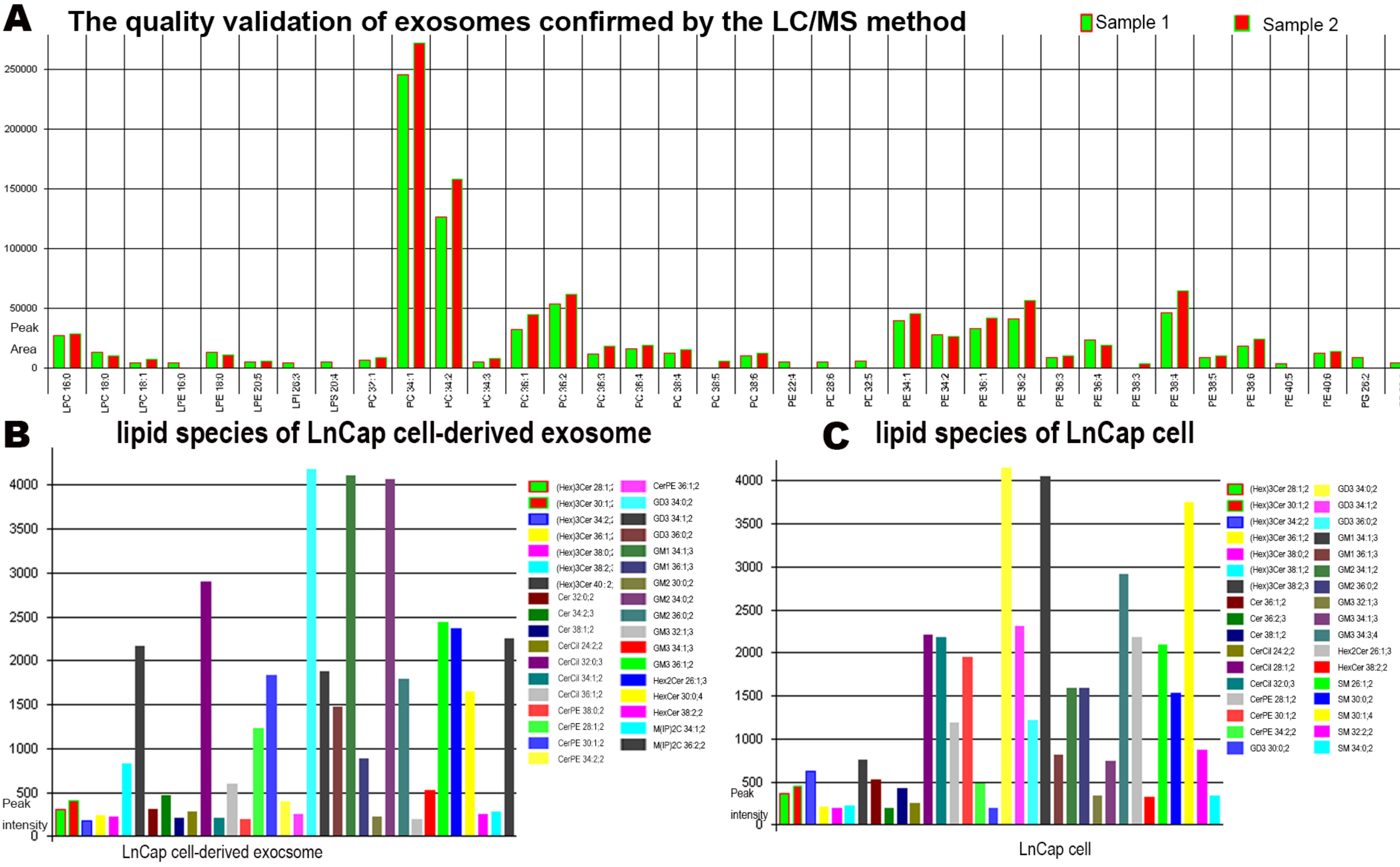

Figure S1

A, Verification of optimized mass spectrometry conditions; B, lipid species of LnCap cell-derived exosomes; and C, lipid species of LnCap cells.

Figure S2: Lipid composition of exosomes of PC3 and DU-145 cells

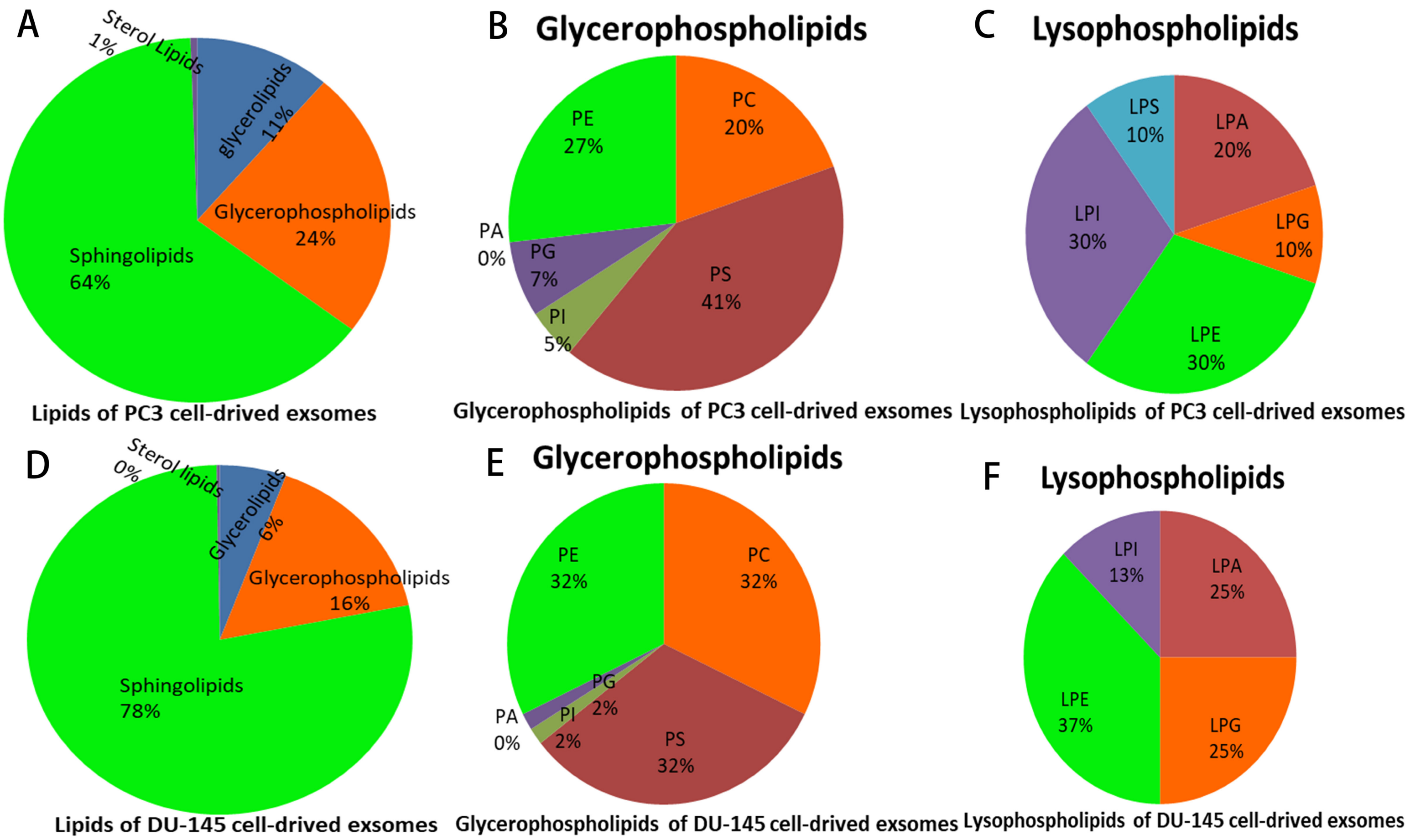

Figure S 2: Lipid composition of exosomes of PC3 and DU-145 cells.

A, Lipid species of PC3 cell-derived exosomes. B, Composition of glycerophospholipids in PC3 cell-derived exosomes. C, Composition of lysophospholipids in PC3 cell-derived exosomes. D, Lipid species of DU-145 cell-derived exosomes. E, Composition of glycerophospholipids in DU-145 cell-derived exosomes. F, Composition of lysophospholipids in DU-145 cell-derived exosomes.

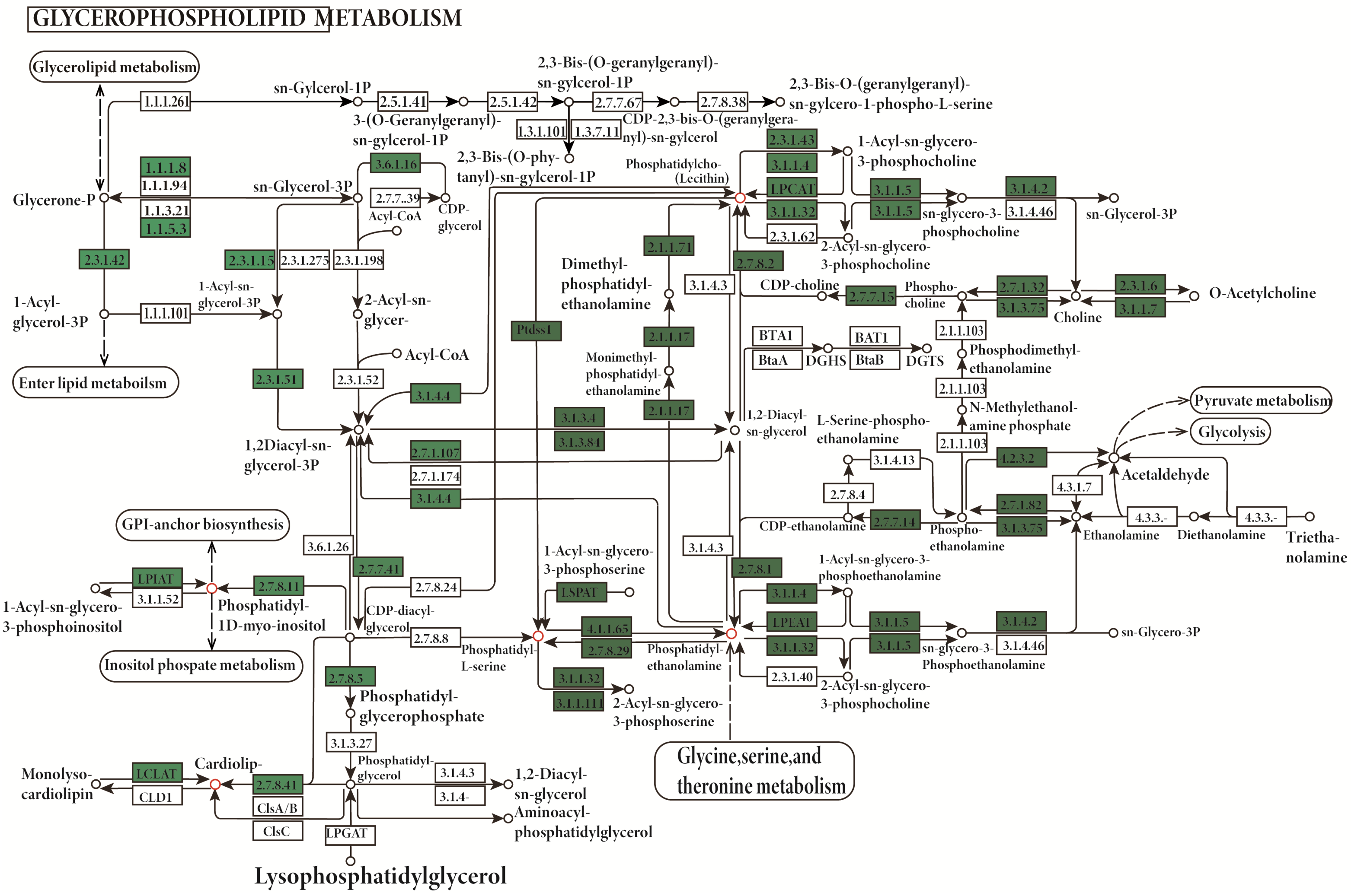

Figure S3

Glycerophospholipid metabolism pathway in phospholipids of exosomes from LnCap cells. Copyright from the Lipid Pathway Enrichment Analysis (LIPEA).

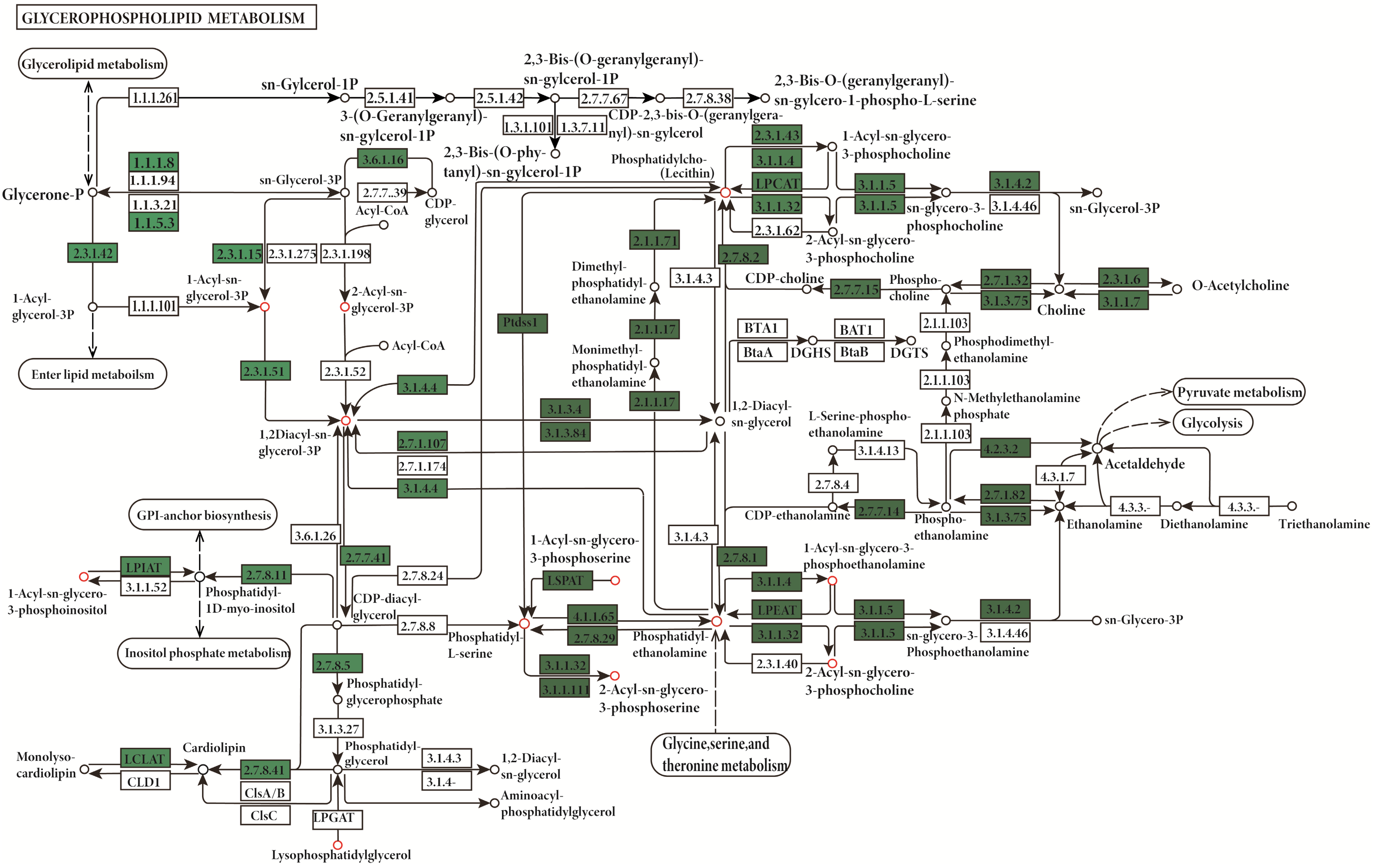

Figure S4

Glycerophospholipid metabolism pathway in phospholipids of exosomes from PC3 and DU-145 cells. Copyright from the Lipid Pathway Enrichment Analysis (LIPEA).

Table S1.

Differences in lipid species between LnCap cells and Lncap cell-derived exosomes.

Supplement Table 1 Differences in lipid species between LnCap cells and Lncap cell-derived exosomes

Lipid type/quantity source	LnCap cells	Exosomes
Cholesterol (CHOL)	1	1
Cholesterol Ester (CE)	7	3
Glyceride diester (DAG)	22	19
Phosphatidylcholine (PC)	28	43
Phosphatidic acid (PA)	1	1
Phosphatidylethanolamine (PE)	31	40
Phosphatidylglycerol (PG)	5	7
Phosphatidylinositol (PI)	4	13
Phosphatidylserine (PS)	27	23
Sphingomyelin (SM)	24	18
Ceramide (CER)	11	11
Hexoside ceramide (HEXCER)	8	8
Lactosylceramide (LACCER)	3	3
Lysophosphatidylcholine (LPC)	0	1
Lysophosphatidylethanolamine (LPE)	2	2
Lysophosphatidylinositol (LPI)	2	2
Phosphatidylglycerol (PG)	5	7
